# Phylotranscriptomics reveals the reticulate evolutionary history of a widespread diatom species complex

**DOI:** 10.1111/jpy.13281

**Published:** 2022-08-08

**Authors:** Ozan Çiftçi, Andrew J. Alverson, Peter van Bodegom, Wade R. Roberts, Adrienne Mertens, Bart Van de Vijver, Rosa Trobajo, David G. Mann, Walter Pirovano, Iris van Eijk, Barbara Gravendeel

**Affiliations:** ^1^ Institute of Environmental Sciences (CML) Leiden University Box 9518 2300 RA Leiden The Netherlands; ^2^ Naturalis Biodiversity Center Darwinweg 2 2333 CR Leiden The Netherlands; ^3^ BaseClear B.V Sylviusweg 74 2333 BE Leiden the Netherlands; ^4^ Department of Biological Sciences University of Arkansas, 1 University of Arkansas Fayetteville Arkansas 72701 USA; ^5^ Diatomella IJkelaarstraat 3 6611 KN Overasselt The Netherlands; ^6^ Meise Botanic Garden Meise, Research Department Nieuwelaan 38 1860 Meise Belgium; ^7^ University of Antwerp, Department of Biology – ECOBE Universiteitsplein 1 B‐2610 Wilrijk Belgium; ^8^ IRTA‐Institute for Food and Agricultural Research and Technology, Marine and Continental Waters Programme Ctra de Poble Nou Km 5.5, E43540, La Ràpita Catalonia Spain; ^9^ Royal Botanic Garden Edinburgh Edinburgh EH3 5LR Scotland UK; ^10^ Bayer Crop Science Leeuwenhoekweg 52 2661 CZ Bergschenhoek The Netherlands; ^11^ Radboud Institute for Biological and Environmental Sciences Heyendaalseweg 135 6500 GL Nijmegen The Netherlands

**Keywords:** cryptic species; diatom, hybridization; incomplete lineage sorting, *Nitzschia palea*, molecular, morphology, phylogeny, reticulate evolution, transcriptomes.

## Abstract

In contrast to surveys based on a few genes that often provide limited taxonomic resolution, transcriptomes provide a wealth of genomic loci that can resolve relationships among taxonomically challenging lineages. Diatoms are a diverse group of aquatic microalgae that includes important bioindicator species and many such lineages. One example is *Nitzschia palea*, a widespread species complex with several morphologically defined taxonomic varieties, some of which are critical pollution indicators. Morphological differences among the varieties are subtle and phylogenetic studies based on a few genes fail to resolve their evolutionary relationships. We conducted morphometric and transcriptome analyses of 10 *Nitzschia palea* strains to resolve the relationships among strains and taxonomic varieties. *Nitzschia palea* was resolved into three clades, one of which corresponds to a group of strains with narrow linear‐lanceolate valves. The other morphological group recovered in the shape outline analysis was not monophyletic and consisted of two clades. Gene‐tree concordance analyses and phylogenetic network estimations revealed patterns of incomplete lineage sorting and gene flow between intraspecific lineages. We detected reticulated evolutionary patterns among lineages with different morphologies, resulting in a putative recent hybrid. Our study shows that phylogenomic analyses of unlinked nuclear loci, complemented with morphometrics, can resolve complex evolutionary histories of recently diverged species complexes.

**Abbreviations**
BLAST: Basic Local Alignment Search Tool
BS: Bootstrap
BUSCO: Benchmarking Universal Single‐Copy Orthologs
EFA: Elliptic Fourier Analysis
gDF: gene discordance factor
ILS: incomplete lineage sorting
LPP: local posterior probability
ML: maximum likelihood
NCBI: National Center for Biotechnology Information
TCC: Thonon Culture Collection

1

Diatoms (Bacillariophyta) are one of the most diverse and abundant groups of microalgae. Their ecological importance and high specificity toward different environmental parameters (e.g., salinity, pH, and nutrients) make diatoms ideal bioindicators (Smol and Stoermer 2010). Various environmental applications, such as water quality biomonitoring and paleoecological reconstructions, rely on assessments of diatom communities (Fritz et al. 1991, CEN 2004). Nearly all these applications are based on species identification using the famously character‐rich and intricately ornamented siliceous cell walls of diatoms, which provide a wealth of characters for taxonomy and species delimitation. Quantitative morphometric methods have also been developed and proved reliable for distinguishing differences among diatom species (Theriot and Ladewski 1986, Pappas et al. 2014).

The DNA sequencing has shown, however, that estimates of diatom diversity based on cell morphology can be highly underestimated (Sarno et al. 2005, Sunagawa et al. 2015). Molecular phylogenetic studies indicate that many widespread diatom morphospecies are comprised of several distinct and often reproductively isolated lineages that are indistinguishable using morphological criteria alone. Examples of these include, *Cyclotella meneghiniana* (Beszteri et al. 2005), *Pseudo‐nitzschia delicatissima* (Quijano‐Scheggia et al. 2009), *Gomphonema parvulum* (Kermarrec et al. 2013), and *Pinnularia borealis* (Pinseel et al. 2019). Moreover, it is estimated that detectable genetic differences can arise between large populations of planktonic diatom species in just 10^2^ years (Lewis et al. 1997, Krasovec et al. 2019), and speciation can take place over periods of 10^3^–10^4^ years or less (Theriot 1992, Mann 1999).

Resolving relationships among recently diverged lineages is essential for improving our understanding of diatom systematics and biodiversity, recovering valuable information about their ecology and evolution, and improving environmental applications that rely on critical bioindicator species. In such cases, however, classical morphological characters traditionally used to separate species can be scarce or uninformative, while the unique features of taxonomically challenging taxa might be physiological or biochemical (Mann et al. 2021). Therefore, molecular phylogenies based on a broader genome sampling may provide better‐resolved evolutionary relationships. Moreover, the expectation for recently diverged species is that speciation history will be reflected by paraphyletic or polyphyletic gene trees, and the use of multiple unlinked loci from the nuclear genome is necessary to account for events such as incomplete lineage sorting (ILS) and gene flow (Mallet 2005, Alverson 2008). Transcriptomes are a valuable resource in this sense because they are relatively inexpensive to obtain, and they can be sequenced without a priori information about the size and complexity of the target genome. Furthermore, tree‐based methods for orthology inference can now accommodate the complex nature of transcriptome data from nonmodel organisms without reference genomes (Yang and Smith 2014, Emms and Kelly 2015, Cheon et al. 2020).

We use this approach to investigate *Nitzschia palea*, a common bioindicator species with global distribution (Finlay et al. 2002). The nominate variety is described as tolerant to heavy metals and an indicator of pollution, whereas *Nitzschia palea* var. *debilis* prefers cleaner waters (Lange‐Bertalot 1980, Van Dam et al. 1994, Sabater 2000, Potapova and Hamilton 2007). However, the type specimens of these varieties show considerable overlap in their morphological characters (Trobajo and Cox 2006). Morphometric, reproductive, and phylogenetic analyses demonstrated that it was impossible to separate the complex into the traditionally recognized varieties, although *N. palea* is a monophyletic group comprised of several distinct evolutionary lineages (Trobajo et al. 2009, Trobajo et al. 2010). Rimet et al. (2014) expanded these studies by including more isolates and detected biogeographic signals but failed to find an objective criterion for marking varietal boundaries.

To date, phylogenetic studies of *Nitzschia palea* were based on *cox*1, *rbc*L, and partial LSU rDNA sequences, and most intraspecific relationships were poorly resolved or unsupported. Although organelle genome sequencing would likely provide many additional phylogenetic markers, sequencing would require genome skimming of a species with an unknown genome size. Moreover, many diatoms cannot grow in the absence of their bacterial microbiomes, so genome skimming would produce a mix of diatom and bacterial sequences, adding further uncertainty to the amount of predicted sequencing effort necessary to obtain large numbers of phylogenetic markers from the target diatom. One advantage of transcriptomics is the ability to select polyadenylated eukaryotic mRNA during library preparation, thereby enriching transcripts from the target diatom genome. This approach has been used successfully to address similar questions across phylogenetic scales in nonmodel plants (Guo et al. 2020, Feng et al. 2021) and animals (Thawornwattana et al. 2018, Williams et al. 2022), and even diatoms (Parks et al. 2017, 2018). Therefore, we hypothesize that genome‐scale analyses based on multiple unlinked nuclear loci will provide a stronger phylogenetic signal with *N. palea*, and provide insights into historical and ongoing processes shaping the evolution of a widespread microbial eukaryote.

We used *Nitzschia palea* as a model to infer the evolutionary history of a large diatom species complex by analyzing morphometric and transcriptomic data from 10 *N. palea* strains to; (i) resolve phylogenetic relationships within the complex, (ii) determine whether morphological characters are congruent with molecular‐based clades, and (iii) discriminate between ILS and gene flow to resolve patterns of reticulate evolution.

## 2 MATERIALS AND METHODS

### 2.1 Sample collection and algal culturing

We acquired 10 *Nitzschia palea* strains from culture collections (Table 1). Four strains (DCG0091, DCG0092, DCG0094, and DCG0751) had been cryopreserved, and three (DCG0091, DCG0092, and DCG0094) were among those studied by Trobajo et al. (2009) (Belgium3, Belgium1, and Belgium2, respectively). Strains TCC13901 and TCC13903 had been included in the study of Rimet et al. (2014). All strains were grown in a WC medium (Guillard and Lorenzen 1972) at 19°C and on a 16:8 h light:dark cycle. We routinely examined the live cultures under a Zeiss Axio Imager M2 microscope (Carl *Zeiss* BV, Breda, The Netherlands).

**Table 1:** Collection, isolation information, and GenBank accessions of *Nitzschia palea* and *Tryblionella levidensis* strains analyzed (BCCM: Belgian Coordinated Collections of Microorganisms, Belgium; TCC: Thonon Culture Collection, France; AJA: Alverson Lab collection).

Strain identifier	Collection	Collection strain identifier	Locality	Isolation date	GenBank Accession no.
DCG0091	BCCM	(02)7D	WWTP^a^, Destelbergen, Ghent, Belgium	July 27, 2005	GJIR00000000
DCG0092	BCCM	(02)9E	WWTP^a^, Destelbergen, Ghent, Belgium	July 27, 2005	GJOZ00000000
DCG0094	BCCM	(02)9F	WWTP^a^, Destelbergen, Ghent, Belgium	July 27, 2005	GJPA00000000
DCG0751	BCCM	16BE	Scheldt river, Ghent, Belgium	December 18, 2016	GJPB00000000
TCC13901	TCC	TCC139‐1	Lake of Geneva, France	November 4, 2009	GJPC00000000
TCC13903	TCC	TCC139‐3	Lake of Geneva, France	December 3, 2010	GJPD00000000
TCC523	TCC	TCC523	River, Saint‐Denis, La Réunion	February 10, 2010	GJPI00000000 GJPH00000000
TCC641	TCC	TCC641	River, Viichtbach, Boevange/Attert, Luxembourg	January 27, 2010	GJPE00000000
TCC852	TCC	TCC852	River, Casal da Misarela, Portugal	April 10, 2013	GJPF00000000
TCC907	TCC	TCC907	Upland stream, Northumberland, UK	January 1, 2015	GJPG0000000
*Tryblionella levidensis*	AJA	AJA077‐10	Lake Ouachita, Arkansas, USA	June 5, 2013	GJPZ00000000

^a^ Wastewater treatment plant.

### 2.2 SEM imaging, morphometrics, and shape outline analysis

For scanning electron microscopy (SEM), we removed organic material by oxidizing the diatoms with hydrogen peroxide and then rinsing them several times with distilled water. Cleaned frustules were dried onto aluminum stubs and coated with platinum‐palladium (Pt 80%, Pd 20%) in a Quorum Q150TS sputter coater (Quorum Technologies Ltd., UK). Four strains (DCG0091, DCG0092, DCG0094, and DCG0751) were observed on a JSM‐6480 Low Vacuum (JEOL) SEM platform at 10 kV, and the remaining strains were observed on a JSM‐7600F Field Emission (JEOL) Scanning Microscope system at 5 kV. We collected 50 images per strain at magnifications ranging from 8000–15,000x. The width (μm), length (μm), stria density (per 10 μm), and fibula density (per 10 μm) of each image were measured or counted using the software package Fiji‐ImageJ (Schindelin et al. 2012). Measurements of stria and fibula densities were made in the center of the valve along its apical axis over 10 μm. Additional descriptive morphological data investigated in previous studies on *Nitzschia palea* were also recorded (Trobajo and Cox 2006). For the analysis of shape outlines, we performed an Elliptic Fourier Analysis (EFA) using the R package Momocs (Bonhomme et al. 2014). EFA is also implemented in diatom morphometric tools such as DiaOutline (Wishkerman and Hamilton 2018) and SHERPA (Kloster et al. 2014). We extracted valve outlines from SEM images using the “Quick Selection” tool of Adobe Photoshop CC 2019 and exported these on a white background. We used the R package Momocs for (i) extracting X and Y coordinates of the valve outline shape, (ii) pre‐processing (i.e., smooth, center, scale, and align), and (iii) computing Elliptic Fourier Transforms (EFT). Finally, we performed a Principal Component Analysis (PCA) analysis using Momocs and visualized the first two principal components (PC). Boxplot and PCA figures were produced using the R package ggplot2 (Wickham and Chang 2016).

### 2.3 RNA extraction and transcriptome sequencing

We extracted total RNA from exponentially growing cultures using the Qiagen RNeasy Plant Mini Kit (Qiagen Benelux BV, Venlo, The Netherlands). First, we removed the excess culture medium and concentrated the cells through repeated centrifugation steps at 2900*g* for 10 min. Final suspensions were transferred to 2 mL tubes containing 0.5 mm zirconia/silica beads (BioSpec, Lab Services BV, Breda, The Netherlands) and the lysis solution provided with the kit. Cells were lysed with a Qiagen Tissue Lyser II (Qiagen Benelux BV, Venlo, The Netherlands) by bead beating for 3 min. Subsequent steps followed the manufacturer's protocol. We assessed the quantity and quality of RNA samples on an Agilent 2100 Bioanalyzer system (Agilent Technologies, Amstelveen, The Netherlands), and RNA integrity (RIN) values ranged from 5.2 to 7.2. Sequencing libraries were prepared using the Illumina TruSeq Stranded mRNA Library Preparation Kit (Illumina, The Netherlands). Sequencing was performed at Baseclear BV (Leiden, The Netherlands) in two separate runs on a NovaSeq 6000 platform (Illumina), generating 2 × 150 bp paired‐end reads. RNA extraction and transcriptome sequencing of the outgroup strain, *Tryblionella levidensis*, followed Parks et al. (2018). Raw sequencing reads were deposited in the Sequence Read Archive (SRA) database of the National Center for Biotechnology Information (NCBI) under BioProject accession PRJNA756685.

### 2.4 Transcriptome assembly

Filtering and assembly of sequencing reads followed Parks et al. (2018) with minor modifications. We inspected the quality of the raw reads using SolexaQA++ (ver. 3.1.7; Cox et al. 2010). Error‐correction was performed using BFC (Li 2015) with parameters: ‐k 31 (k‐mer length) and ‐s 50. Sequencing reads were trimmed using Trimmomatic (ver. 0.39; Bolger et al. 2014) with the options ‘ILLUMINACLIP:2:30:10 LEADING:3 TRAILING:3 SLIDINGWINDOW:4:12 MINLEN:50’. As the final step of the pre‐assembly process, we removed all reads mapping to plastid and mitochondrial genomes of *Nitzschia palea* (strain NIES‐2729; Kamikawa et al. 2018; AP018511 and AP018512, respectively) and the SILVA rRNA database using bowtie2 (ver. 2.4.1) in “‐‐very‐sensitive‐local” mode (Langmead and Salzberg 2012). The filtered reads were assembled using Trinity (ver. 2.10) with strand‐specific options (Grabherr et al. 2011). We assessed assembly quality by checking the recovery of conserved eukaryotic orthologs in the BUSCO database (ver. 4; Waterhouse et al. 2018). Assembled nuclear transcripts were translated into amino acid sequences with TransDecoder (ver. 5.5.0; Haas 2020) with guidance from BLASTP (ver. 2.8.1) searches against the SwissProt database (The Uniprot Consortium 2019) with an e‐value cut‐off of 1e^−3^, and HMMER (Finn et al. 2011) searches against the Pfam database (El‐Gebali et al. 2019). Redundant transcripts were filtered using CD‐HIT (ver. 4.8.1; Li and Godzik 2006) with a sequence identity threshold of 0.99 and a word length of five. Finally, we quantified transcript abundances using Salmon (ver. 1.0.0; Patro et al. 2017) and selected the most highly expressed isoform per trinity gene for downstream analyses using the respective utility script from Trinity with the “‐‐highest‐iso‐only” option.

### 2.5 Orthology inference and species tree reconstruction

We used OrthoFinder (ver. 2.4.0; Emms and Kelly 2019) to construct putative orthologous clusters from the complete set of predicted proteins. Building and pruning homolog trees for species tree reconstruction followed the *phylogenomic data set construction* pipeline of Yang and Smith (2014). Briefly, we extracted coding sequences for orthogroups that contained at least one transcript per strain, aligned the amino acid sequences with MAFFT (ver. 7.47; Katoh and Standley 2013), and trimmed alignments with Phyx (Brown et al. 2017) using a minimal column occupancy threshold of 0.2. Orthogroup gene trees were constructed using RAxML (ver. 8.2.12; Stamatakis 2014) with the GTRCAT model and 100 rapid bootstrap replicates. Outlier long branches were removed with TreeShrink (Mai and Mirarab 2018), and mono‐ and paraphyletic tips that belonged to the same strain were pruned, along with deep paralogs, using respective Python scripts from Yang and Smith (2014). For each strain, the sequence (a terminal branch, or ‘tip’, on the gene tree) with the most unambiguous characters in the trimmed alignment was retained, and each reduced orthogroup was realigned with MAFFT. These amino acid alignments were used to guide codon alignments with pal2nal (Suyama et al. 2006). Codon alignments were trimmed using Phyx as described above, and maximum likelihood homolog trees were inferred using IQ‐TREE 2 (Minh et al. 2020a) with 100 bootstrap replicates and the TESTMERGE procedure with codon position partitions specified for each alignment. TESTMERGE selects the best‐fit partitioning scheme followed by model selection and tree construction. As the last step of the pruning pipeline, we extracted one‐to‐one orthologs from these bootstrapped homolog trees using Yang and Smith's (2014) MI strategy with a long internal branch cut‐off of 0.6. Ortholog trees were inferred using IQ‐TREE 2 as described above. Finally, we estimated two species trees using summary‐coalescent and concatenation‐based approaches. We used ASTRAL‐III (Zhang et al. 2017) with the set of unrooted ortholog trees to estimate the first species tree and local posterior probability (LPP) support values. The concatenated ML analysis was performed using IQ‐TREE 2 with 100 bootstrap replicates to estimate branch support. The model of sequence evolution for each locus was calculated using ModelFinder, as implemented in IQ‐TREE 2, with separate substitution models and separate evolutionary rates across sites. A summary workflow for all steps from data collection to species tree estimation is given in Figure S1 in the Supporting Information. *Tryblionella levidensis* was specified as the outgroup strain in the ASTRAL‐III analysis, and the ML species tree was rooted manually using FigTree (ver. 1.4.4).

### 2.6 Quantifying genealogical concordance

Gene concordance factors were computed using IQ‐TREE 2 by comparing the alternative resolutions of quartets of taxa around each branch (Minh et al. 2020b). The gene concordance factor (gCF) describes the proportion of concordant gene trees with a given split in the species tree, whereas the proportions of concordant gene trees supporting the two alternative topologies are reported as gene discordance factors, gDF_1_ and gDF_2_. The proportion of all other conflicting resolutions, which result in different arrangements of paraphyletic gene trees, is reported as the third gene discordance factor, gDF_P_. Under strict ILS assumptions and ignoring gene tree error, gene trees supporting the two alternative topologies (gDF_1_ and gDF_2_) are expected to occur in roughly equal frequency (Huson et al. 2005), and the significance of this prediction was tested using a chi‐squared test. Unequal proportions of these two alternative topologies are expected when certain sequences are more closely related to the members of one neighbor branch than the other, which might indicate gene flow between clades (Huson et al. 2005). Gene concordance and discordance factors were mapped onto the species tree as pie charts, and the mean shapes for each strain were illustrated at the tips using the R packages ape (Paradis et al. 2004) and ggtree (Yu et al. 2017).

### 2.7 Phylogenetic network estimations and tests for introgression

We estimated phylogenetic networks while accounting for ILS and gene flow using two approaches to explore intraspecific relationships in *Nitzschia palea*. First, we estimated a phylogenetic network under maximum pseudo‐likelihood using the InferNetwork_MPL command in PhyloNet (Yu and Nakhleh 2015, Wen et al. 2018). We used the complete set of rooted gene trees, applied a bootstrap support threshold of 95%, ran ten independent network searches, and sequentially tested the allowance of 0 to 3 hybridization/reticulation branches. We selected the run with the highest log pseudo‐likelihood as the best estimate. We visualized PhyloNet results using Dendroscope (Huson et al. 2007). A sharp improvement in score is expected until it reaches the best value and has a slower, linear improvement after that. Second, we estimated a phylogenetic network using the NANUQ algorithm (Allman et al. 2019). We used all gene trees and ran NANUQ through the MSCquartets R package (Rhodes et al. 2021). We used a small alpha (0.01) and a large beta (0.95) for hypothesis testing following the developer's recommendations. We used the test results on the quartet counts from all gene trees to calculate a network distance matrix between taxa. We then inferred a split network from the distance matrix using the Neighbor‐Net algorithm (Huson and Bryant 2006). As a complement to the network approaches, we also performed introgression tests for the putative hybrid (TCC907) with Patterson's *D*‐statistic (Durand et al. 2011), commonly known as the ABBA‐BABA test. The ABBA‐BABA test is based on site patterns in the alignments under a null ILS model (i.e., no gene flow). Given the genome sequences of three ingroups and an outgroup population with the relationship (((P1,P2),P3),O), ABBA sites are those at which P2 and P3 share a derived allele (B), while P1 has the ancestral state (A). The BABA pattern represents sites at which P1 and P3 share the derived state. The ABBA‐BABA test approximates the proportion of the genome represented by these two discordant topologies. In the absence of any deviation from a strict bifurcating topology (i.e., no gene flow), we expect to find roughly equal proportions of ABBA and BABA site patterns in the genome. The *D*‐statistic test is used to quantify deviations from this proportion. We calculated *D* using the calcD function in the R package evobiR (Blackmon and Adams 2015) and assessed the significance of *D* using a block jackknife approach (block‐size 1000 sites, 1000 replicates).

## 3 RESULTS

### 3.1 Morphometric data and valve outlines

**Fig. 1:**
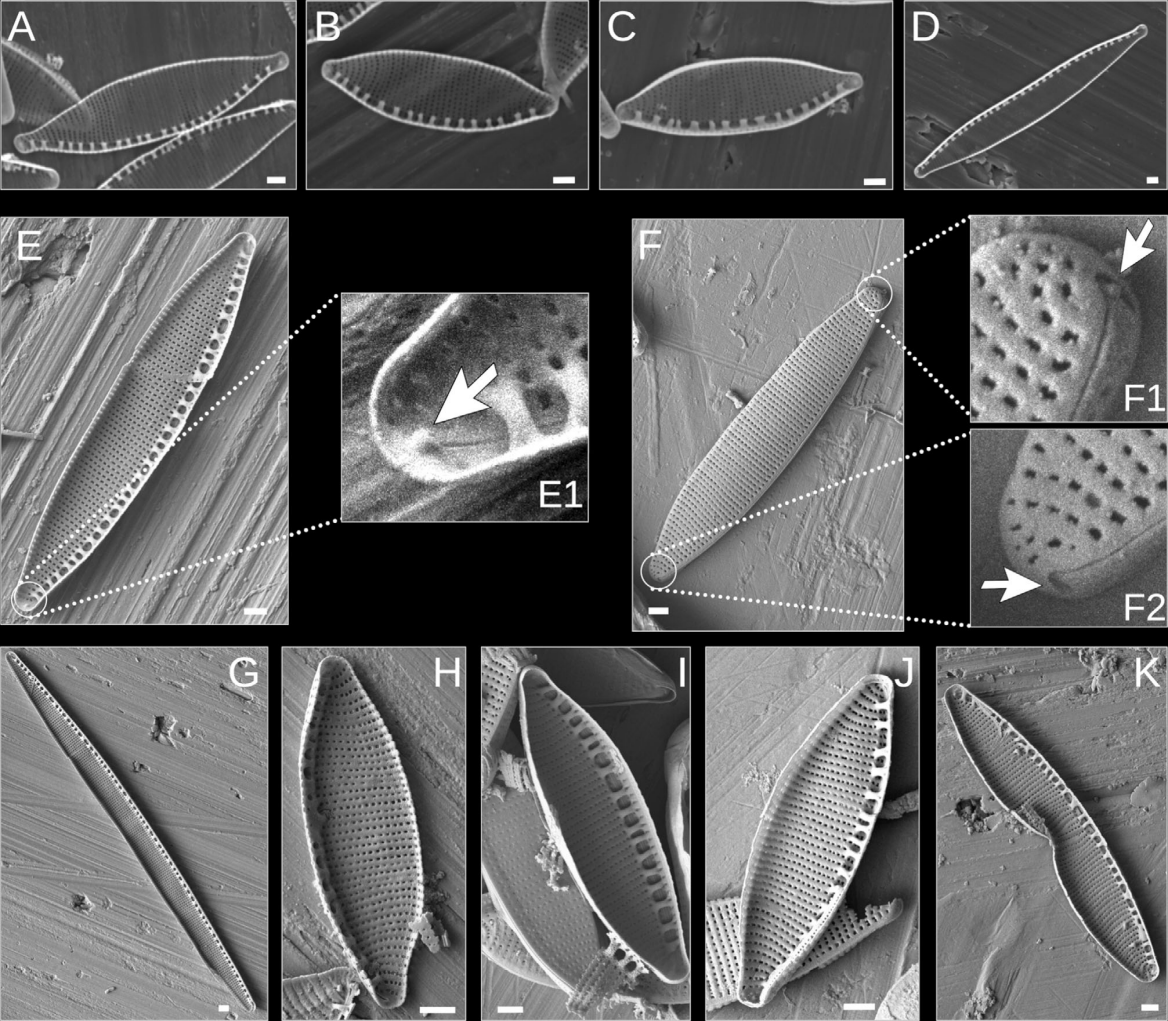
Scanning electron micrographs of *Nitzschia palea* strains analyzed in this study. (A) DCG0091, (B) DCG0092, (C) DCG0094, (D) DCG0751, (E) TCC13091, (E1) Lower apex of TCC13901 showing helictoglossa, (F) External view of TCC13901, (F1) Upper apex of TCC13901 showing the polar raphe ending, (F2) Lower apex of TCC13901 showing the polar raphe ending, (G) TCC13903, (H) TCC523, (I) TCC641, (J) TCC852, (K) TCC907, representing a teratological form. All scale bars equal 1 μm.

The valves that we examined with SEM had the following morphological features: (i) linear‐lanceolate to lanceolate outlines with rostrate to subcapitate apices, (ii) irregularly spaced fibulae with median fibulae not more widely spaced, (iii) no central raphe endings, (iv) terminal raphe fissures that internally end in helictoglossae (Fig. 1, E1), and (v) polar raphe endings that turn toward the same side in a single valve (Fig. 1, F1 and F2). These characters agree well with the analysis of the type material of *Nitzschia palea* from the Kützing collection at the Natural History Museum, London, UK (Trobajo and Cox 2006). The only exceptions were valves with deformed features.

**Fig. 2:**
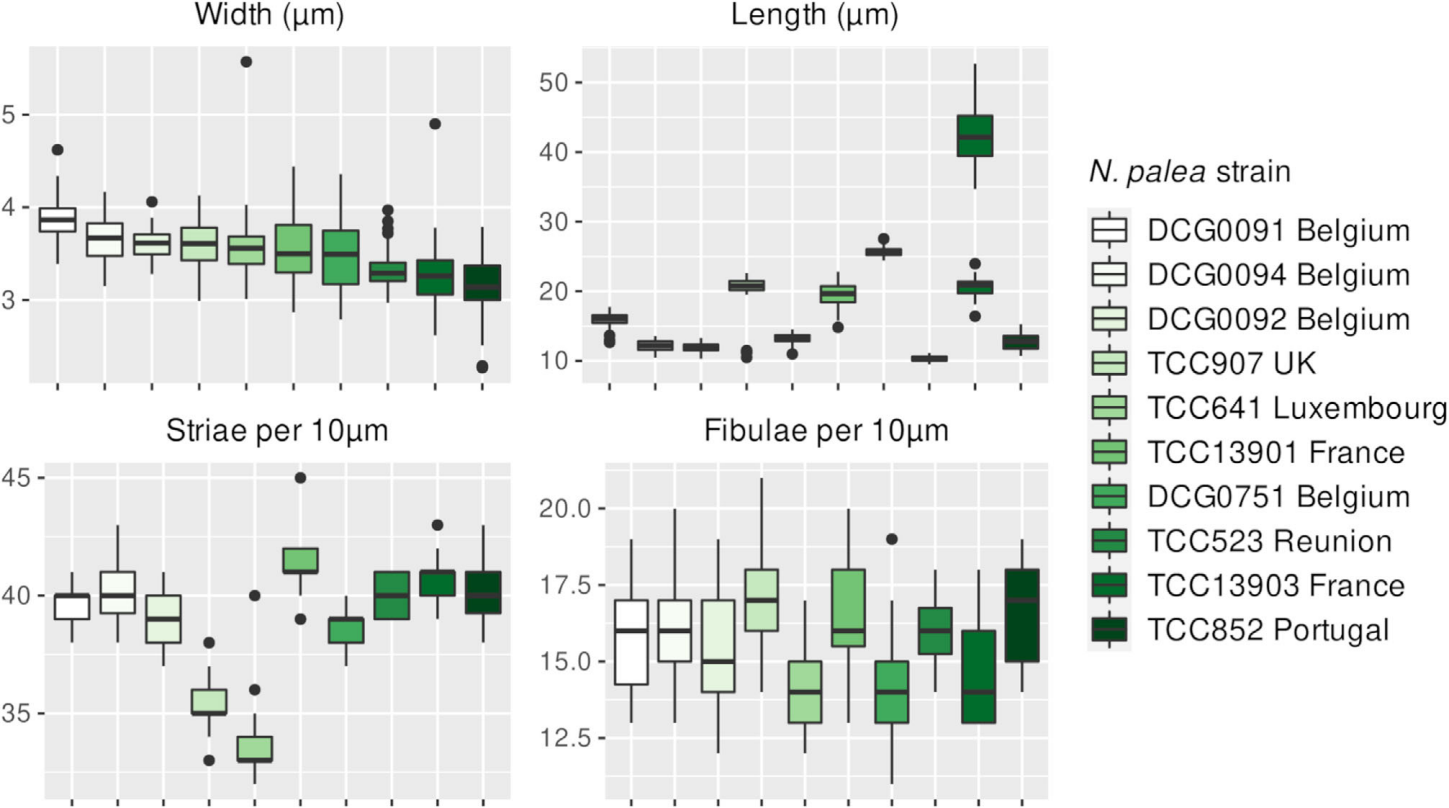
Boxplot summary of morphological measurements of *Nitzschia palea*. Black dots represent outliers. Strains are ordered based on their mean width.

The observed morphometric measurements, summarized in Figure 2, overlap with the ranges indicated for the types of *Nitzschia palea* (Trobajo and Cox 2006), except for deformed valves and the mean lengths of five strains that were small due to size reduction caused by long‐term cultivation (Fig. 1K; Table S1 in the Supporting Information). Specifically, most of the cells in strain TCC907 had stria and fibula deformations and retractions at the center of the valve margins on one side. We removed this strain from the shape outline analysis and only used its valve lengths and widths. Many valves of TCC641 and TCC523 had stria and fibula deformations, and we measured a minimum of 30 valves for these two strains. The number of images used in the EFA for TCC641 and TCC852 was lower due to valve outline deformities (Table S2 in the Supporting Information). The deformations of TCC907 and TCC641 are apparent in Figure 2, with low stria densities that fall out of the ranges described from the type specimens (Table S1; Trobajo and Cox 2006). We observed two different length ranges (greater than 34.5 μm and less than 25 μm) for TCC13903 (Fig. 2). This strain is known to be able to complete its life cycle and restore large size in monoclonal culture (F. Rimet, pers. comm.); i.e., it is unlike some of the clones studied by Trobajo et al. (2009) and Bagmet et al. (2020), which were shown to be incapable of sexual reproduction without a compatible partner (i.e., they were heterothallic). Its stria density varied the least among all strains analyzed, providing corroborative evidence for the presence of a single strain with two different length ranges, possibly representing different stages of its growth cycle (Table S1).

**Fig. 3:**
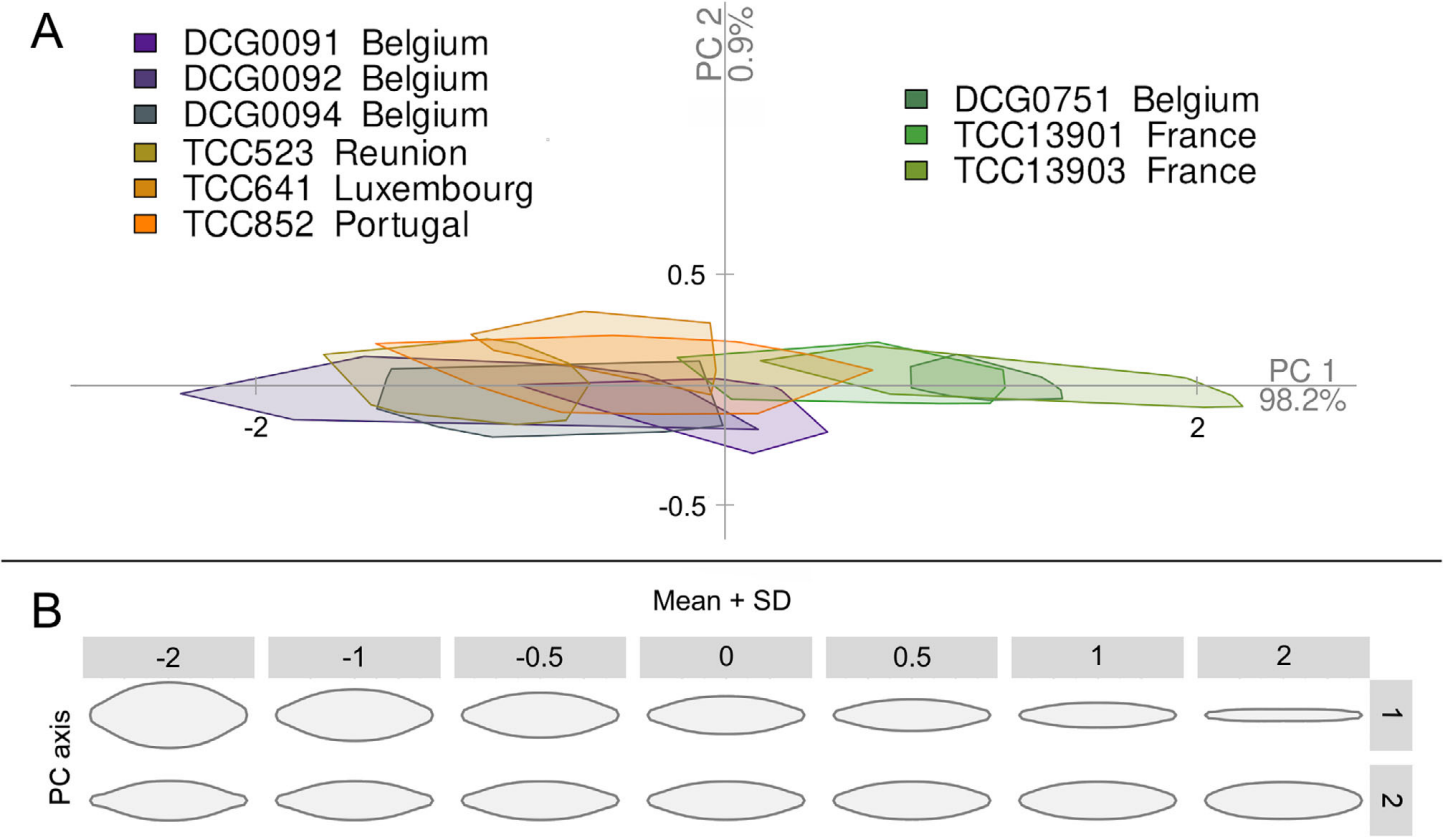
(a) PCA results of *Nitzschia palea* valve outline analysis. (b) Shape variation along the first two principal component axes. Standard deviations were calculated as the square roots of the eigenvalues of the covariance matrix.

Based on the morphological criteria used in Trobajo et al. (2009), none of the strains in this study can be assigned to *Nitzschia palea* var. *debilis* (i.e., valve width <3.5 μm and stria density > 43 per 10 μm). Four strains (DCG0751, TCC523, TCC13903, and TCC852) were narrower than 3.5 μm. However, the highest mean stria density in this group was 40.7 per 10 μm (TCC13903; Table S1). Two of these four narrow strains (TCC523 and TCC852) had many deformities. The strain with the largest mean stria density in our sample set was TCC13901 (41.2 per 10 μm) with a mean width of 3.5 μm. The first principal component explained 98.2% of the total variation in valve shape (Fig. 3A) and showed a gradient from lanceolate to linear‐lanceolate outlines (Fig. 3B). Two narrow strains without deformities (TCC13903 and DCG0751) and the strain with the largest mean stria density (TCC13901) overlapped at the linear‐lanceolate end of the morphospace (Fig. 3A). The remaining six strains included in the shape outline analysis (TCC523, TCC641, TCC907, DCG0091, DCG0092, and DCG0094) were much shorter than the ranges described for *N. palea*, probably due to size reduction caused by long‐term cultivation. DCG0092 had the lowest mean stria and fibula densities in this group, whereas TCC852 had the largest (Fig. 2). These six strains were positioned at the lanceolate end of morphospace in shape outline analysis, showing a slight overlap with the linear‐lanceolate group (Fig. 3A). The second principal component in the shape outline analysis explained less than 1% of the total variation in valve shape outline (Fig. 3A) but showed a gradient from subcapitate to rounded ends (Fig. 3B). All strains overlapped considerably based on this axis, and there was no apparent clustering.

### 3.2 Transcriptome assemblies, orthology inference, and species tree estimation

**Fig. 4:**
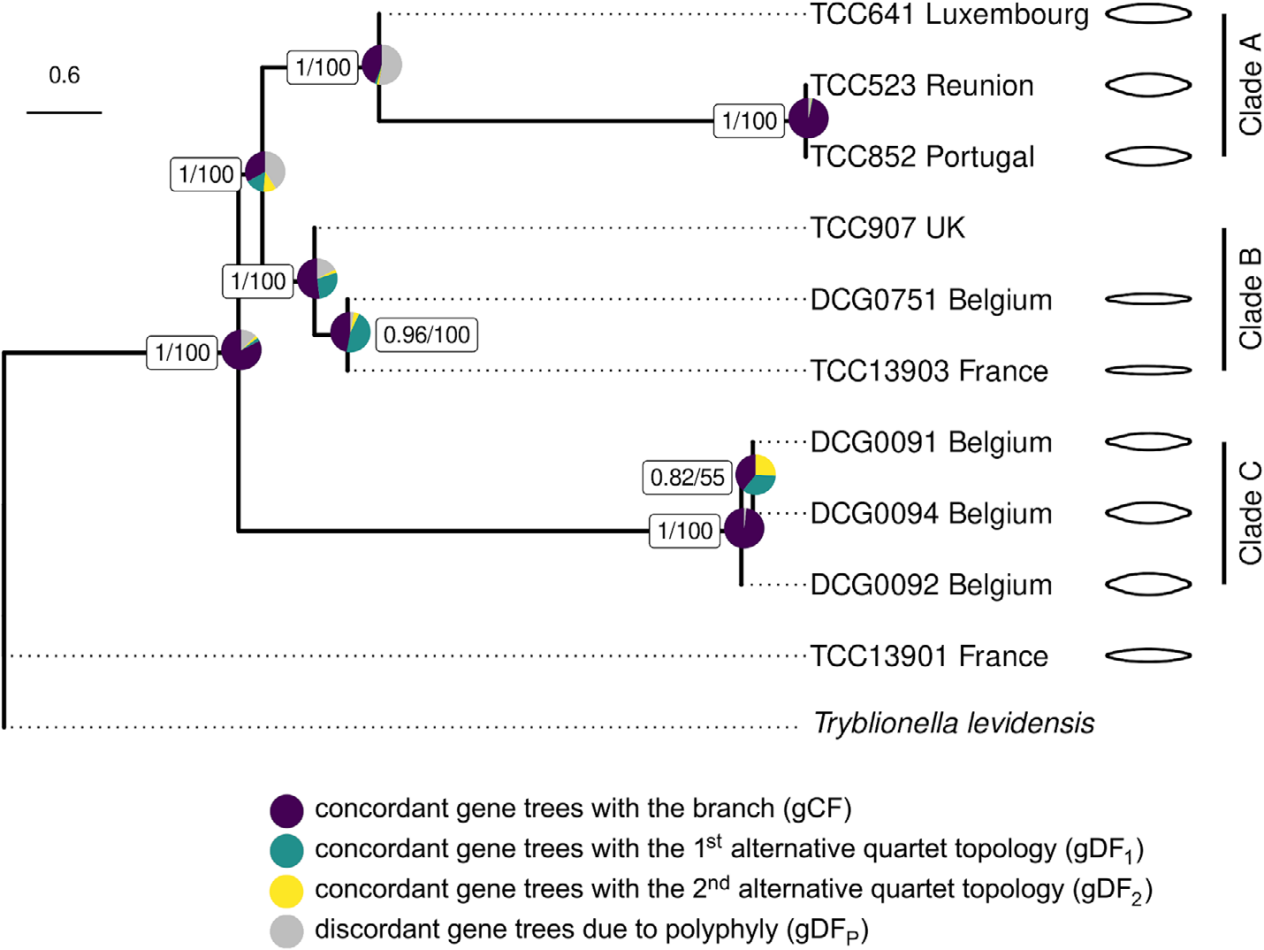
ASTRAL‐III species tree estimated from 183 orthologs. Pie charts show IQ‐TREE 2 gene concordance and discordance factors. ASTRAL‐III LPP values (left) and bootstrap support from concatenated ML analysis (right) are given in boxes. Branch lengths are in coalescent units. Dotted lines are used to align the tip labels. Mean shapes from EFA for each strain are illustrated at the tips. No outline image is given for TCC907, which had deformed outlines representing an artificial shape due to culture conditions.

We assembled transcriptomes for 10 *Nitzschia palea* strains and an outgroup, *Tryblionella levidensis*. Datasets ranged in size from 15.6 to 33.4 million reads per strain. Two sets of reads were assembled for strain TCC523, with duplicates removed during the tree pruning procedure. Trinity assemblies ranged in size from 23,791 to 110,789 genes and 34,388 to 157,240 transcripts, including isoforms. The average BUSCO recovery of the assembled transcriptomes was 74 ± 14%, indicating that we captured a large portion of the gene space in each strain. After filtering to remove transcripts with low expression levels, the final data set used for ortholog clustering contained 16,109–62,583 Trinity transcripts per strain. Among the 81,016 total orthogroups identified by OrthoFinder, 1260 of them included data for all ten strains. We obtained 515 homolog alignments with full taxon occupancy, estimated trees from these orthogroups, cut aberrantly long internal branches, and removed paralogs by trimming and masking monophyletic tips from the same strain (Yang and Smith 2014). Using Yang and Smith's (2014) MI strategy, a final tree‐pruning step to select orthologs resulted in 183 one‐to‐one ortholog trees with alignment lengths ranging from 396 to 4935 bp. The total concatenated alignment length for these 183 orthologs was 283,107 bp, where 137,025 (48.4%) of these sites were invariant, 65,405 (23.1%) were parsimony informative, and 80,677 (28.5%) were variable but parsimony uninformative. The topologies of ASTRAL and ML species trees were identical. Three main clades (Fig. 4, clades A–C) were recovered with maximum LPP and BS values (LPP = 1, BS = 100) with both methods (Fig. 4). All other branches in the estimated species trees, except for the internal branch in Clade C (LPP = 0.8, BS = 55), were strongly supported (LPP >0.96, BS = 100).

### 3.3 Gene tree concordance analyses, phylogenetic network inferences, and tests for introgression

Among recently diverged taxa, individual gene trees can disagree with the underlying species tree due to ILS or gene flow (Maddison 1997, Degnan and Rosenberg 2006). We obtained maximum support values for the relationships among the three main clades in the species trees, despite evidence for widespread ILS in the evolutionary history of the *Nitzschia palea* species complex (Fig. 4). In our gene tree concordance analysis, only 61 of the 183 gene trees supported a sister relationship of clades A and B (Fig. 4). The proportions of gene trees with alternate topologies gDF_1_ (15%) and gDF_2_ (11%) were not significantly different for this branch (Fig. 4), consistent with ILS. A large proportion of the discordance for this relationship was due to paraphyly (41%), which comprises gene trees where one or more clades around this branch are not monophyletic. The split between TCC641 and the other two Clade A strains also was poorly supported (gDF_P_ = 52.5%). Deeper in the species tree, a large majority (151) of the gene trees supported a sister relationship of Clade C to the common ancestor of clades A and B (Fig. 4). Chi‐squared tests for unequal gDF_1_ and gDF_2_ values were significant (*P* < 0.05) only for the two branches in Clade B, suggestive of a process other than ILS underlying discordance at this branch.

**Fig. 5:**
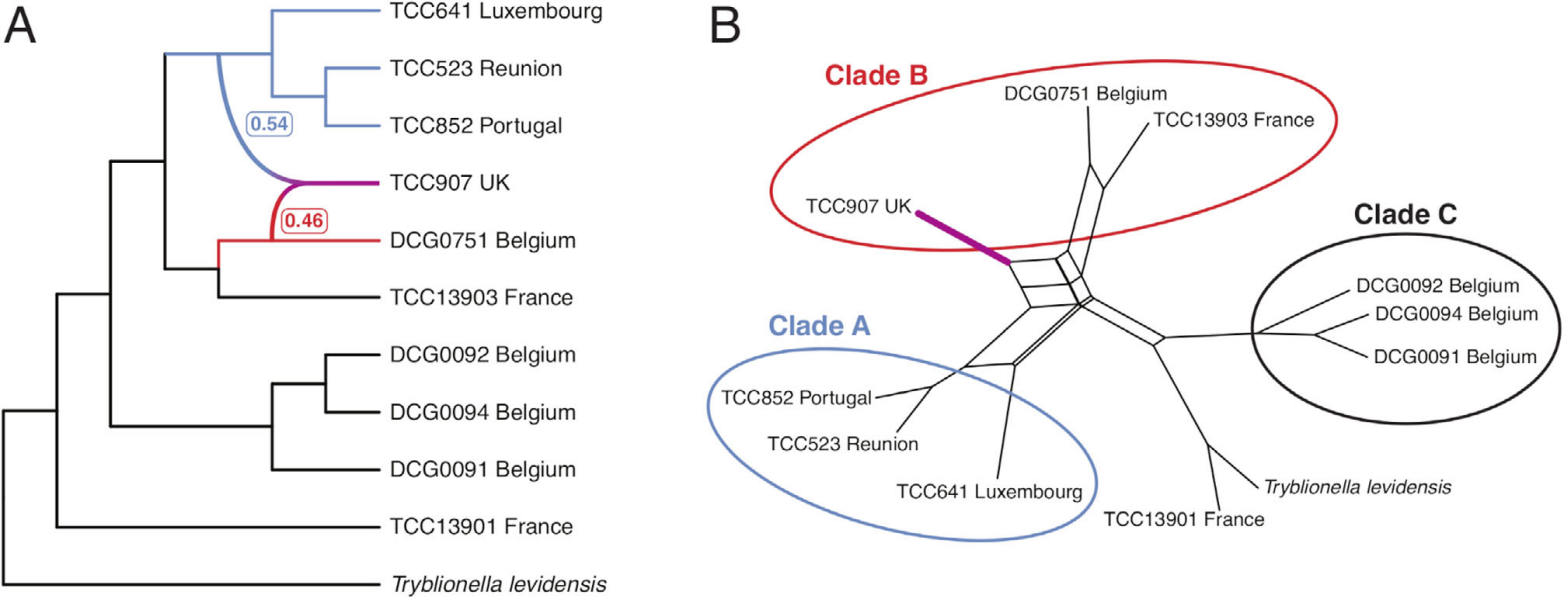
Phylogenetic networks estimated using (a) PhyloNet with inheritance probabilities (ɣ) from parent lineages of the putative hybrid indicated in *Nitzschia palea*, and (b) hybrid detection with NANUQ. Clade labels correspond to those recovered in the species tree (Fig. 4).

We detected large proportions of gene trees concordant with the first alternative topology for both nodes in Clade B (Fig. 4, green vs. yellow), consistent with gene flow between Clade A and Clade B populations. The phylogenetic trees inferred by ASTRAL and ML were based on a model of strict bifurcation. To test for the possibility of a more complex network model, we used three different methods. First, we used PhyloNet to estimate the best network from the set of 183 gene trees. This analysis identified TCC907 in Clade B as a putative hybrid between Clade A and DCG0751 (Fig. 4, Clade B) with near‐equal inheritance probabilities (ɣ; Fig. 5A). To further test the hypothesis that TCC907 is of hybrid origin, we used NANUQ and MSCQuartets to estimate a phylogenetic network, and this analysis revealed further evidence for reticulation between Clade A and Clade B populations, with strain TCC907 placed as an intermediate between these parent clades (Fig. 5B). Finally, we performed the ABBA‐BABA test for the putative hybrid, TCC907, using the concatenated alignment of 183 orthologs where three Clade A strains were specified as one parental population and the other two members of Clade B as the second. Our results revealed a substantial excess of ABBA site patterns with a significant positive *D*‐statistic regardless of the order of parental populations, further corroborating the results from the network analyses (D: 0.6/0.9; Z‐score: 87/630).

## 4 DISCUSSION

This study aimed to resolve intraspecific relationships within the widespread bioindicator diatom species, *Nitzschia palea*. There is considerable overlap in the morphological characters of the varieties described from waters with different pollution levels (*N. palea* and *N. palea* var. *debilis*), making the species complex taxonomically challenging (Trobajo and Cox 2006, Trobajo et al. 2009). Using phylotranscriptomics, we resolved the *N. palea* species complex into three monophyletic groups. None of these clades contained strains that could be assigned to *N. palea* var. *debilis* based on the criteria used in Trobajo et al. (2009). However, two narrow strains without deformities (DCG0751 and TC13903) and the strain with the largest mean stria density (TCC13901) were recovered at the linear‐lanceolate end of the morphospace in shape outline analysis. These strains partly meet the criteria or fall closest to the ranges that are given by Trobajo et al. (2009). Both species trees recovered two linear‐lanceolate strains (DCG0751 and TCC13903) and TCC907 in Clade B with maximum support values, though as discussed in more detail below, TCC907 is likely of recent hybrid origin. The third linear‐lanceolate strain (TCC13901) was recovered outside of the three main clades in both species trees and has the largest mean stria density, providing additional evidence for both morphological and phylogenetic differentiation of this strain (Fig. 2; Table S1). Strain TCC13903 in Clade B can restore its large size, indicating that there might be variation in the reproductive characteristics within the *N. palea* species complex, as demonstrated for other diatom species as well (Round et al. 1990).

The remaining six *Nitzschia palea* strains fell into two distinct clades (Fig. 4, Clades A and C). The morphometric measurements for strains in both clades overlapped with the ranges indicated for the nominate variety of *N. palea* (Table S1), which includes populations with medium to large valve width and are most abundant in more polluted freshwater habitats (Trobajo and Cox 2006, Hofmann and Werum 2017). In the shape outline analysis, we recovered these strains at the lanceolate end of morphospace, though they overlap slightly with the linear‐lanceolate Clade B strains (PC1; Fig. 3). Three strains in Clade C represent sympatric clones isolated simultaneously from a wastewater treatment plant in Belgium, and these are members of one mating type recognized previously as ‘*N. palea* mating group 1’ by Trobajo et al. (2009). Our stria and fibula measurements for these strains are slightly higher than those given by Trobajo et al. (2009), possibly due to the effects of size reduction in culture (Table S1). On the gradient of valve end shapes, three Clade C strains had the most subcapitate ends in our sample set based on their mean shapes (PC2; Fig. 3). However, all three Clade A strains had many deformed valves and missing data, so it is not easy to compare these two groups based on morphometric data alone. Moreover, broader morphometric ranges for the characters measured here have been recorded from other clones of *N. palea* (Bagmet et al. 2020), highlighting that the full range of morphological variation present in this species complex was not represented by the limited number of strains in this study, and many more populations need to be sampled for discussion of *N. palea* morphology. Nonetheless, our species tree estimations recovered ‘*N. palea* mating group 1’ as sister to the common ancestor of Clade A and Clade B, whereas the remaining three lanceolate strains (Clade A) were recovered as sister to the linear‐lanceolate Clade B strains, with maximum support values for both relationships (Fig. 4). In conclusion, in agreement with the previous studies, the clades that we recovered did not show a clear morphological distinction as one linear‐lanceolate strain (TCC13901) was recovered as a distinct lineage from the linear‐lanceolate Clade B, and the two lanceolate clades (A and C) were not monophyletic.

We cannot provide any conclusions on the ecological variability of the *Nitzschia palea* species complex with current data. It is necessary to integrate experimental and field observations with our approach to reveal any physiological and ecological differences which might exist between the clades recovered in this study. Similarly, more populations need to be sampled to capture the full array of genetic variation in this species complex.

When reconstructing the evolutionary history of a lineage, ILS and gene flow are two main causes of incongruence between the species tree and individual gene trees. ILS occurs when lineages have not been reproductively isolated for long enough for ancestral polymorphisms to fully assort in the descendant lineages so that the gene trees are not reciprocally monophyletic and the speciation history cannot be reflected accurately (Avise 1990). For very recently diverged species, such as the populations of *Nitzschia palea* studied here, the probability of ILS is higher due to the persistence of ancestral polymorphisms that can reduce phylogenetic signal (Maddison 1997). Unlike concatenation‐based species tree estimation, gene tree summary methods are more robust to discordance between gene trees and species trees in the presence of ILS (Mirarab et al. 2014). Sampling error is reduced in large phylogenomic datasets usually resulting in increased branch support, but the probability of violating the underlying assumptions in phylogenomic inference, such as homogeneity across sites and evolutionary time, is also higher (Kumar et al. 2012). Therefore, capturing the underlying agreement and disagreement of gene trees in phylogenomic datasets requires additional methods (Kumar et al. 2012, Minh et al. 2020b). Consistent with these expectations, we observed evidence of widespread ILS in the evolutionary history of the *N. palea* species complex using gene concordance factors, and the estimated species trees showed very high support for all branches, except for one internal branch (Fig. 4, Clade C). Across our species tree, the highest proportions of gene trees that provided evidence of ILS were for Clade C, which is expected as these are members of a single interfertile population. Our findings highlight that complementary approaches to gene tree summary methods are necessary to quantify the extent of discordance in the evolutionary history of recently diverged species complexes.

Gene flow among lineages due to hybridization is another process that can generate gene tree discordance and distort phylogenetic signal across the genome (Slatkin and Maddison 1989). Phylogenomic analyses of gene flow have to account for ILS as both processes can generate discordance between gene trees and species trees (Hibbins and Hahn 2022). Models of a strictly bifurcating tree can be rejected in favor of a network model that hypothesizes reticulation events to account for instances of gene tree discordance (Wen et al. 2018). Using phylogenetic network methods, we obtained further insights into the relationships between the three clades recovered in the species trees, revealing reticulated evolutionary patterns between lanceolate Clade A and linear‐lanceolate Clade B populations (Fig. 5B). Although we recovered clear phylogenetic separation of these two populations with high statistical support, the mating systems (e.g., cell recognition) in these two lineages apparently have not diverged enough to prevent gene flow. Previous studies, using controlled crosses between clones in laboratory conditions, showed some degree of reproductive isolation between groups of clones (Trobajo et al. 2009), but such crossing tests, though informative, need to be complemented by genetic evidence such as we provide here. Further sampling from natural populations will show the full extent of gene flow in this species complex and whether there is a biogeographic component.

Hybridization between intraspecific diatom lineages has been reported for *Seminavis robusta* (de Decker et al. 2018) and *Eunotia bilunaris* (Vanormelingen et al. 2008) based on evidence from crossing experiments and between two varieties of *Pseudo‐nitzschia pungens* based on evidence from morphological and phylogenetic analyses (Casteleyn et al. 2009). There were also earlier reports of hybridization from natural populations between morphological variants within nominal species (summarized by Mann 1999). Our study presents the first report of hybrid detection in a diatom species complex based on genome‐scale data. Although the parentage cannot be conclusively identified for this putative hybrid given our current sampling, its genome is presumed to be allodiploid, as the inheritance probabilities from Clade A and DCG0751 were nearly equal. Evidence for allopolyploidy has also been reported in the diatom *Fistulifera solaris* based on genome‐wide heterozygosity patterns (Tanaka et al. 2015).

Despite limited strain sampling, the phylotranscriptomic approach used here increased the number of genes used for phylogenetic inference by greater than 60‐fold compared to previous phylogenetic studies of the *Nitzschia palea* species complex. Our matrix included >280,000 characters (bp). Previous investigations of *N. palea* used some combination of plastid *rbc*L (1334 bp), nuclear LSU rDNA (498 bp), and mitochondrial *cox*1 (495 bp; Trobajo et al. 2009, 2010). Although these three markers provided some resolution within the complex, most relationships were poorly supported and deeper relationships remained unresolved (Rimet et al. 2014). We used data from 183 unlinked nuclear loci and obtained maximum support values for most intraspecific relationships. But more importantly, our data provided new insights into mechanisms of evolutionary change in *N. palea*. For example, our data set revealed a large degree of gene‐tree discordance, suggesting a recent divergence of this species complex that may not be accurately estimated with standard molecular clock approaches and highlighting that studies based on one or a few markers are not guaranteed to recover the organismal phylogeny. The recent study of *N. palea* by Bagmet et al. (2020) illustrates this differently: eight of the nine original clones they studied were shown to be reproductively compatible, producing a viable F1 (the ninth was incapable of sexual reproduction because of its small size), but these eight fell into two widely separated groups based on partial *cox*1 sequences, differing from each other by a remarkable 73 substitutions (in 679 aligned bp). Genome‐scale analyses of unlinked nuclear genes also allowed us to identify a putative hybrid resulting from recent gene flow between lineages with different morphologies. The DNA sequencing has led to the splitting of once broadly defined widespread diatom taxa into several species some or all of which were subsequently found to differ morphologically (e.g. Sarno et al. 2005), but our findings indicate that gene flow might obscure species boundaries in taxa that are very recently diverged. Some species complexes are quite old (e.g., the *Sellaphora pupula* complex; Evans et al. 2008) and are more likely to be fully reproductively isolated. Ours is one of a growing number of phylogenomic studies in nonmodel organisms that has greatly increased the amount of phylogenetic signal compared to the use of few standard markers and revealed patterns of gene‐tree discordance due to biological phenomena such as ILS, hybridization, and polyploidy (Smith et al. 2015, Thawornwattana et al. 2018, Williams et al. 2022).

Our analyses revealed a complex evolutionary history of the *Nitzschia palea* species complex. We resolved the species complex into three clades, one of which corresponded to a group of narrow linear‐lanceolate strains. However, one other linear‐lanceolate strain was recovered outside of the three main clades in the phylogenetic analyses, indicating that the linear‐lanceolate morphology might have originated multiple times in this species. Although our sampling of the geographic and morphological diversity within *N. palea* was limited, the phylotranscriptomic approach here nevertheless provided a strong phylogenetic signal and new insights into the evolution of *N. palea*. Studies of other widespread diatom species will show whether similar processes are at play in those as well. Our study demonstrates that phylotranscriptomics provides an efficient and cost‐effective way to resolve such patterns.

## AUTHOR CONTRIBUTIONS

**O. Çiftçi:** Conceptualization (equal); data curation (lead); formal analysis (equal); investigation (lead); methodology (equal); resources (equal); software (equal); visualization (equal); writing – original draft (lead). **A. J. Alverson:** Conceptualization (equal); formal analysis (equal); methodology (equal); resources (equal); software (equal); visualization (equal); writing – review and editing (equal). **P. Van Bodegom:** Supervision (equal); writing – review and editing (equal). **W. R. Roberts:** Formal analysis (equal); methodology (equal); software (equal); visualization (equal); writing – review and editing (equal). **A. Mertens:** Conceptualization (equal); investigation (equal); methodology (equal); writing – review and editing (supporting). **B. Van de Vijver:** Conceptualization (equal). **R. Trobajo:** Methodology (equal); writing – review and editing (equal). **D. G. Mann:** Methodology (equal); writing – review and editing (equal). **W. Piruvano:** Project administration (equal); supervision (supporting); writing – review and editing (supporting). **I. Van Eijk:** Formal analysis (supporting); investigation (supporting). **B. Gravendeel:** Funding acquisition (lead); project administration (lead); resources (equal); supervision (equal); writing – review and editing (equal).

## Supporting information


**Figure S1.** Summary of bioinformatic workflow. Green boxes show the input and output of each step with the file format specified below. Light orange boxes follow the tools and information on the version used.

**Table S1.** Mean value and SD of morphometric measurements per strain compared with type slides of *Nitzschia palea*. Ranges for the measurements are given at the bottom of each cell. Strains are ordered based on their width.

**Table S2.** The number of valves used in shape outline analysis of *Nitzschia palea* per strain and the number of valves removed from the data set due to tilting caused by stacking on SEM stubs and valve outline deformations.
