# Supplementary material for: Phylotranscriptomics reveals the reticulate evolutionary history of a widespread diatom species complex
Source: J Phycol. 2022 Aug 8;58(5):643–56. doi: 10.1111/jpy.13281 (PMC9804273; doi:10.1111/jpy.13281)
Supplement: Supplementary file 2 — Table S1. Mean value and SD of morphometric measurements per strain compared with type slides of Nitzschia palea. Ranges for the measurements are given at the bottom of each cell. Strains are ordered based on their width. [file JPY-58-643-s001.docx]

Table S1. Mean value and SD of morphometric measurements per strain compared with type slides of *Nitzschia palea*. Ranges for the measurements are given at the bottom of each cell. Strains are ordered based on their width.

| Strain identifier | Origin | Length (µm) | Width (µm) | L - W ratio | Striae  (10µm) | Fibulae (10µm) |
| --- | --- | --- | --- | --- | --- | --- |
| DCG0091 | Belgium | 15.95±1.13  12.7-17.8 | 3.89±0.27  3.4-4.6 | 4.12±0.44 | 39.56±1.09  38-41 | 16.06±1.86  13-19 |
| DCG0094 | Belgium | 12.12±0.87  10.5-13.6 | 3.68±0.25  3.2-4.2 | 3.31±0.35 | 40.38±1.29  38-43 | 15.82±1.34  13-20 |
| DCG0092 | Belgium | 11.92±0.66  10.3-13.3 | 3.6±0.16  3.3-4.0 | 3.31±0.24 | 39.2±1.12  37-41 | 15.42±1.85  12-19 |
| TCC907 | UK | 20.23±2.6  10.5-22.6 | 3.58±0.27  3.0-4.1 | 5.69±0.9 | 35.4±0.91  33-38 | 16.89±1.54  14-21 |
| TCC641 | Luxembourg | 13.15±0.77  11.0-14.6 | 3.56±0.39  3.0-4.0 | 3.72±0.36 | 33.69±1.37  32-36 | 14.23±1.31  12-17 |
| TCC13901 | France | 19.57±1.72  14.8-22.8 | 3.53±0.35  2.9-4.4 | 5.61±0.81 | 41.19±0.97  39-42 | 16.6±1.64  13-20 |
| DCG0751 | Belgium | 25.62±0.69  24.4-27.6 | 3.49±0.37  2.8-4.4 | 7.43±0.82 | 38.76±0.92  37-40 | 14.08±1.56  11-19 |
| TCC523 | Réunion | 10.33±0.41  9.5-11.2 | 3.33±0.21  3.0-4.0 | 3.12±0.25 | 40±0.82  39-41 | 16.14±1.13  14-18 |
| TCC13903 | France | 33.06±11.5  16.4-52.7 | 3.27±0.35  2.6-3.8 | 10.33±3.99 | 40.68±0.67  39-43 | 14.59±1.4  13-18 |
| TCC852 | Portugal | 12.8±1.17  10.7-15.3 | 3.15±0.35  2.3-3.8 | 4.12±0.72 | 40.26±1.11  38-43 | 16.78±1.53  14-19 |
| *N.palea* type* | Germany | 28.06±4.0  25.5-34.0 | 3.83±0.25  3.6-4.17 | unknown | 40.55±2.42  37.4-44.6 | 16.08±1.65  13.9-17.6 |
| *N. palea* var. *debilis* type* | Germany | 28.5±1.3  26.3-30.3 | 3.4±0.2  3.0-3.8 | unknown | 41.4±1.5  39.8-40.0 | 14.8±1.1  13.2-16.4 |

*** Trobajo & Cox (2006) - SEM measurements
