# Supplementary material for: Phylotranscriptomics reveals the reticulate evolutionary history of a widespread diatom species complex
Source: J Phycol. 2022 Aug 8;58(5):643–56. doi: 10.1111/jpy.13281 (PMC9804273; doi:10.1111/jpy.13281)
Supplement: Supplementary file 3 — Table S2. The number of valves used in shape outline analysis of Nitzschia palea per strain and the number of valves removed from the data set due to tilting caused by stacking on SEM stubs and valve outline deformations. [file JPY-58-643-s003.docx]

Table S2. The number of valves used in shape outline analysis of *Nitzschia palea* per strain and the number of valves removed from the dataset due to tilting caused by stacking on SEM stubs and valve outline deformations.

| Strain identifier | Origin | # of outlines analyzed | # of tilted valves | # of deformed outlines |
| --- | --- | --- | --- | --- |
| DCG0091 | Belgium | 43 | 1 | 0 |
| DCG0092 | Belgium | 50 | 0 | 0 |
| DCG0094 | Belgium | 46 | 1 | 0 |
| DCG0751 | Belgium | 48 | 1 | 0 |
| TCC13901 | France | 50 | 2 | 0 |
| TCC13903 | France | 52 | 1 | 0 |
| TCC523 | Réunion | 51 | 0 | 0 |
| TCC641 | Luxembourg | 36 | 2 | 0 |
| TCC852 | Portugal | 38 | 1 | 7 |
| TCC907 | UK | 0 | 5 | 54 |
